# Effect of Ketamine on Cardiovascular Function During Procedural Sedation of Adults

**DOI:** 10.7759/cureus.14228

**Published:** 2021-03-31

**Authors:** Kara Goddard, Christopher Sampson, Starr-Mar'ee Bedy, Rugheed Ghadban, Julie Stilley

**Affiliations:** 1 Emergency Medicine, University of Missouri School of Medicine, Columbia, USA; 2 Cardiology, Washington University School of Medicine, St. Louis, USA

**Keywords:** ketamine, sedation, ischemia

## Abstract

Introduction: Ketamine is commonly used in emergency department procedural sedation. Mild to moderate transient increases in blood pressure, heart rate, and cardiac output are common due to ketamine causing an increase in sympathetic activity. There is a concern that these physiological changes could result in an increased myocardial oxygen demand that may exacerbate underlying cardiac disease.

Methods: Convenience sample of patients older than 50 years receiving ketamine for procedural sedation in the emergency department was used (n = 31). Patients were selected to receive ketamine based on provider discretion. Primary outcome was incidence of new myocardial ischemia apparent on an electrocardiogram (ECG). ECGs were obtained prior to sedation and during the sedation approximately one minute after administration of ketamine. ECGs were reviewed by a board-certified emergency medicine physician and a board-certified cardiologist.

Results: New onset ischemia was found in 9.7% (3/31) of ECGs. Of these, one was in a patient who had previously received ketamine without evidence of ischemia on the repeat ECG. There were no statistically significant differences between the groups. Evidence of ischemia on ECG did not impact patient disposition.

Conclusions: Ketamine is a useful medication in procedural sedation; however, careful attention should be made in patient selection when ketamine is the desired agent. Consideration might be made in using the lowest possible dose of ketamine to obtain adequate sedation in order to hopefully lessen the occurrence of ECG changes suggestive of myocardial ischemia. Based on this small sample, single-site study, no evidence of statistically or clinically significant ischemia was seen with the use of ketamine for procedural sedation. Ketamine remains a safe medication option in adults undergoing procedural sedation.

## Introduction

Ketamine is an agent commonly used in emergency department procedural sedations due to its anesthetic and analgesic properties and respectable safety profile. Mild to moderate transient increases in blood pressure, heart rate, and cardiac output are common due to ketamine’s increase in sympathetic activity. Often this is a desirable effect of ketamine that may help to avoid peri-procedural hypotension. However, there is a concern that these physiological changes could result in an increased myocardial oxygen demand that may exacerbate underlying cardiac disease. Avoidance is recommended for patients with known coronary artery disease, older adults with risk factors for coronary artery disease, or those who are already hypertensive or tachycardic [1].

The incidence of myocardial ischemia following ketamine administration is unknown. Ketamine is included in the American Heart Association (AHA) list of medications that may cause or exacerbate heart failure and has been reported to precipitate myocardial ischemia in the elderly [2,3]. There is limited evidence of ketamine precipitating myocardial ischemia during short-term use in the emergency department (ED).

Emergence reaction can also occur with ketamine administration. Although these reactions can be quickly treated with a benzodiazepine, hypertension and tachycardia are often seen during these episodes, along with hallucinations and panic. This can cause an increase in oxygen consumption, as well, that could attribute to observed cardiac effects, specifically in patients with underlying cardiac disease [4-6].

We sought to discover if patients greater than 50 years of age who received ketamine during routine procedural sedation would have changes suggestive of cardiovascular ischemia seen on an ECG performed during the sedation.

## Materials and methods

The study took place at an academic medical center with a level-one trauma designation that serves as a regional referral center for orthopedic injuries and other specialty care. The annual ED volume is 55,000 patients per year.

Prospectively, a convenience sample of patients older than 50 years receiving ketamine for procedural sedation in the ED was used. Recruitment occurred during hours when the three-person research team members were working clinically in the ED. Patients were selected to receive ketamine based on provider discretion. Patients were offered enrollment after sedation choice was made by the treating provider, and informed consent was obtained if patients agreed to enrollment. Ketamine was not required to be the sole agent used and could be administered with other sedating and analgesic agents. Patients were eligible for enrollment during each visit in the study period. Patients were excluded if they were pregnant, incarcerated, or unable to provide informed consent due to a language barrier or mental status. An initial goal of 50 patients was set, but following two years of recruitment, the study was closed to new subjects. This study had local Institutional Review Board approval.

Primary outcome was the incidence of new changes suggestive of myocardial ischemia apparent on ECG. Ischemia was defined using the third universal criteria from the Task Force for the Universal Definition of Myocardial Infarction (Table 1) [7]. Secondary outcomes included changes in vital signs after ketamine administration and a case-control analysis comparison of patients with changes suggestive of ischemia to those without. Clinically significant vital sign change was defined as an increase or decrease of greater than or equal to 20% from baseline. The Charlson Comorbidity Index (CCI) was used to assess health status. The CCI predicts the 10-year survival in patients with multiple comorbidities.

**Table 1:** Electrocardiogram Manifestations of Acute Myocardial Ischemia Source: Ref. [7].

Myocardial Ischemia Manifestations
ST elevation
New ST elevation at the J point in two contiguous leads with cut-points: ≥0.1 mV in all leads other than leads V_2_-V_3 _where the following cut-points apply: ≥0.2 mv in men ≥ 40 years; ≥0.25 mV in men < 40 years, or ≥0.15 mV in women
ST depression and T wave changes
New horizontal or down-sloping ST depression ≥ 0.05 mV in two contiguous leads and/or T inversion ≥ 0.1 mV in two contiguous leads with prominent R wave or R/S ration ≥ 1

Protocol

Sedation protocol was decided by the physician primarily managing patient's care and was not influenced by the research team. All sedations had a nurse present for the sedation and a physician responsible for monitoring the patient separate from the procedural physician. All patients received continuous monitoring of blood pressure, heart rate, oxygen saturation, and end-tidal CO_2_.

ECGs were obtained prior to sedation and during the sedation approximately one minute after administration of ketamine. One minute was chosen as evaluation time, given the onset of intravenous ketamine being estimated at 30 seconds. ECGs were only obtained for the purpose of the study and were not standard of care at this institution for patients undergoing a procedural sedation. ECGs were obtained on Mortara ELI 350 and ELI 380 machines (Mortara Instrument Inc., Milwaukee, WI). ECGs were reviewed by the physician performing the sedation during acute care for any abnormal findings. Due to the real-time interpretation, any abnormal ECG findings were addressed during the ED visit with any necessary follow-up evaluations ordered. For study analysis, ECGs were reviewed by a board-certified emergency medicine physician and a board-certified cardiologist. ECGs were reviewed for any ischemic changes from the baseline to post-ketamine ECG, with the two ECGs directly compared to one another. Patient demographics, blood pressure, heart rate, and comorbidity data were retrospectively collected from the electronic medical record. Vital signs were reviewed from start to end of the sedation. Vital signs were not available to blinded ECG interpreters.

## Results

Consent was obtained during 33 independent patient encounters, and two patients were removed from the study after enrollment. The subjects were removed due to a change in sedation medication or the decision to forego procedural sedation. There were 31 ECGs included in the final evaluation, with two patients enrolled on subsequent visits. The overall study cohort had a median age of 63 years, with orthopedic manipulation as the most common indication for sedation (Table 2). Of note, all patients who consented to study enrollment were White. This was due to random chance and not due to specific patient selection. Post-ketamine vital signs showed a notable change in 29% (9/31) of initial readings and 71% (22/31) of readings at any point during the sedation.

**Table 2:** Patient Characteristics * Due to the low sample size, ranges are reported in place of IQR. SD, Standard deviation; IQR, interquartile range; BMI, body mass index; HR, Heart rate; SBP, systolic blood pressure; DBP, diastolic blood pressure.

	No Ischemia (N = 28)	Ischemia* (N = 3)	Total (N = 31)
Sex: female, n (%)	15 (53.6)	3 (100)	18 (58.1)
Age, years ± SD	63 (56-70)	64 (63-64)	63 (57-70)
Race: White	28 (100)	3 (100)	31 (100)
Weight in kg (IQR)	86 (75-101)	74 (72-95)	86 (74-99)
BMI	29.2 (24.4-36.6)	30 (27.3-32.8)	28.7 (24.2-35)
Ketamine dose, mg/kg (IQR)	0.56 (0.5-0.82)	0.84 (0.77-0.93)	0.57 (0.5-1)
Sedation indication (%)			
Orthopedic manipulation	23 (82.1)	2 (66.7)	25 (80.6)
Chest tube placement	2 (7.1)	1 (33.3)	3 (9.7)
Burn	2 (7.1)	0	2 (6.5)
Endoscopy	1 (3.6)	0	1 (3.2)
Charlson Comorbidity Index (IQR)	2 (1-4)	2 (2-6)	2 (1-4)
Baseline vitals			
HR, bpm (IQR)	80 (71-86)	85 (78-86)	80 (73-86)
SBP, mmHg (IQR)	134 (126-164)	169 (152-182)	137 (128-169)
DBP, mmHg (IQR)	81 (75-96)	97 (82-106)	82 (76-97)

**Figure 1:**
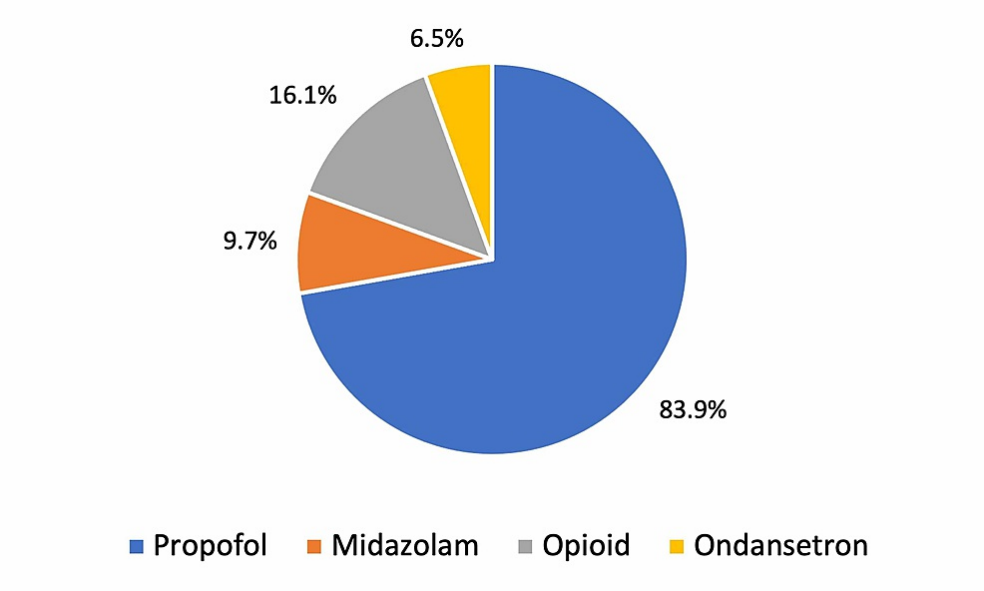
Medications Administered With Ketamine

As noted previously, each patient in the study received dose as part of their procedural sedation. However, it was not a requirement for ketamine to be the sole sedating agent during the sedation. Other agents, including propofol, opioid analgesics, and anti-emetics, specifically ondansetron, were administered during the procedural sedations at the discretion of the treating provider (Figure 1).

**Figure 2:**
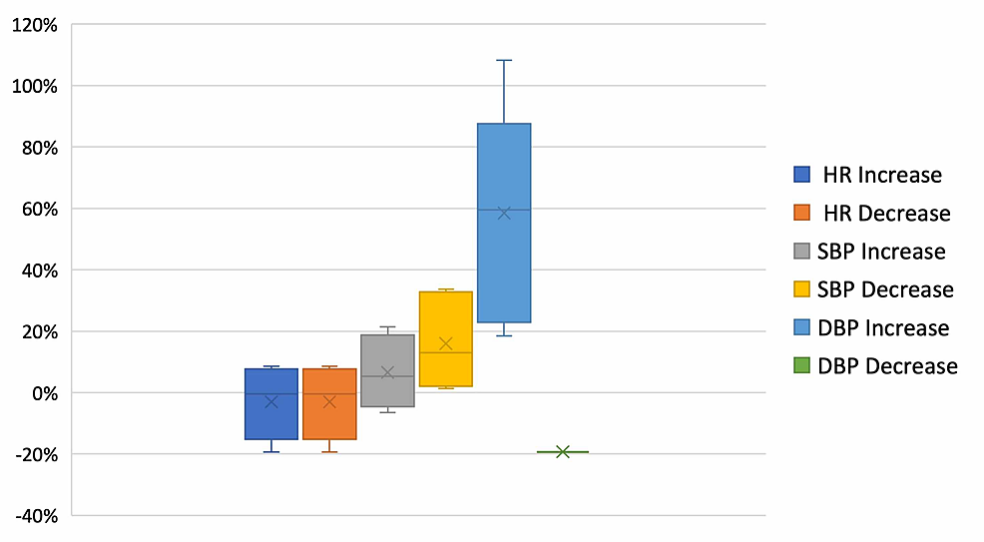
Variability of Vital Signs During Sedation HR, Heart rate; SBP, systolic blood pressure; DBP, diastolic blood pressure.

Figure 2 represents the change in heart rate and blood pressure from the patients’ pre-procedure baseline (indicated as 0% on the graph). Changes suggestive of ischemia including were found in 9.7% (3/31) of ECGs. Two of the ischemic changes on ECG were noted to be ST depression, and one was noted to be T wave inversions. Of these, one was in a patient who had previously received ketamine without evidence of ischemia on the repeat ECG. There were no statistically significant differences between the groups. Evidence of ischemia on ECG did not impact patient disposition. One of the three patients who experienced changes suggestive of ischemia experienced >20% increase from baseline in both blood pressure and heart rate. The other two patients experienced 10%-15% change in blood pressure from baseline, with one of those patients also experiencing 10% change in heart rate from baseline.

**Figure 3:**
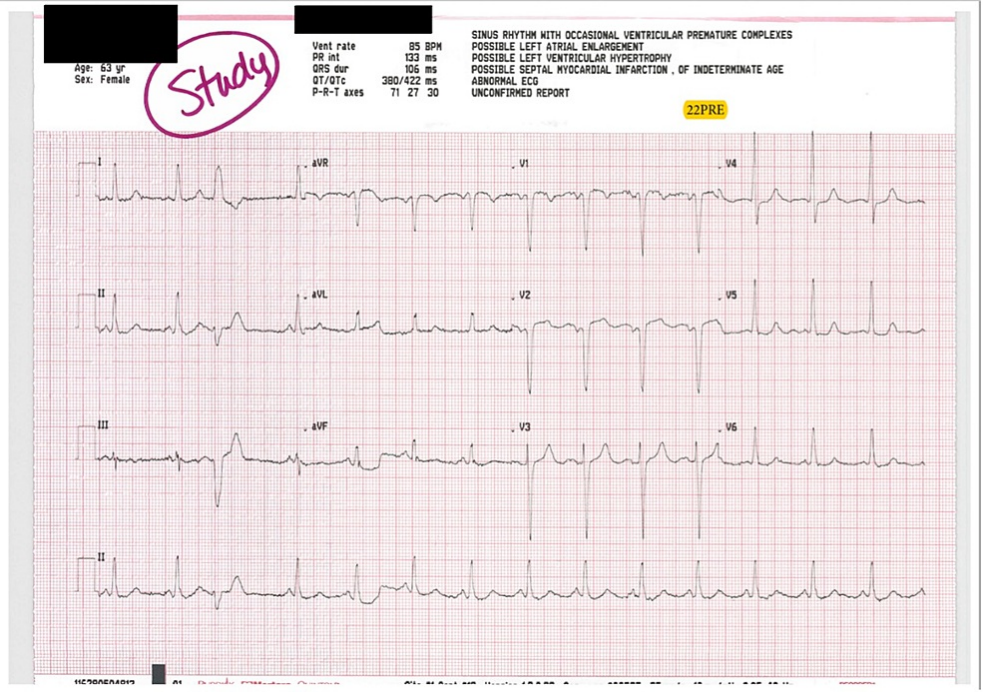
Baseline ECG on Second Visit ECG, Electrocardiogram.

**Figure 4:**
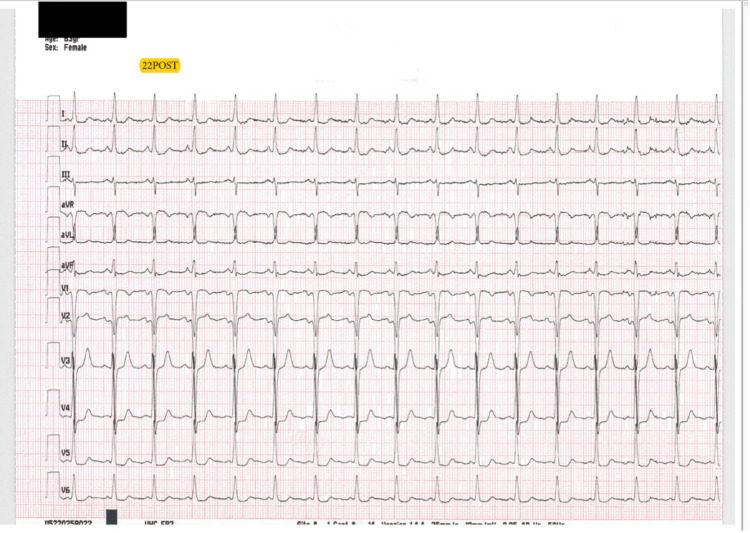
Post-ketamine ECG With Ischemia Present in Leads V4 and V5 ECG, Electrocardiogram.

Overall, the patients received a median dose of 0.57 mg/kg of ketamine. In the ischemia group, the median dose was higher than the milligram per kilogram dosing of the non-ischemia group (0.84 vs. 0.56). One patient in the ischemia group had been enrolled in this study previously on a separate patient encounter. During the initial visit, the patient received 0.56 mg/kg of ketamine and had no myocardial ischemia on the post-ECG; however, during the second encounter, the patient received a slightly higher dose of ketamine (0.69 mg/kg) and had subsequent ischemia present on the post-ECG (Figures 3, 4).

## Discussion

Procedural sedation for various indications commonly takes place in the ED setting. Common dosing for ketamine when given intravenously for procedural sedation ranges from 0.25 mg/kg to 1 mg/kg, depending on if other anesthetic and/or analgesic medications are concomitantly administered. Myocardial ischemia has been reported for ketamine [2,8]; however, it is unclear how soon after medication administration that this could result, namely in the immediate, post-administration timeframe (specifically one minute post administration).

In looking at the incidence of new changes suggestive of myocardial ischemia apparent on ECG immediately following administration of ketamine, it is interesting that an occurrence rate of almost 10% was shown in this study with such a small sample size. It is also notable that all of the patients who experienced ischemia were females. In terms of significant changes in vital signs, it is noteworthy that a significant change (≥20%) from baseline in initial readings occurred in 29% of patients immediately after medication administration, while 71% of patients had a significant change in vital signs at any point during the procedure.

The patients enrolled were overall healthy. Based on the CCI for 10-year survival likelihood, 71% had a score of 3 or less, which correlates to a survival likelihood of 77% or more (score of 3 = 77%, 2 = 90%, 1 = 96%, 0 = 98%); 29% of patients had a score of 4 or more correlating to a 10-year survival likelihood of 53% or less (score of 4 = 53%, 5 = 21%, 6 = 2%).

A major limitation of the study was the use of a convenience sample to recruit subjects. The small research team limited enrollment numbers and generalizability. Another limitation was possible effects of other medications used in the sedation. Timing of ECG obtained during sedation likely had some variability with attempt made to adhere to the one-minute post ketamine administration time frame.

## Conclusions

Ketamine is a useful medication in procedural sedation; however, careful attention should be made in patient selection when ketamine is the desired agent. Consideration might be made in using the lowest possible dose of ketamine to obtain adequate sedation in order to hopefully lessen the occurrence of ECG changes suggestive of myocardial ischemia. Based on this small sample, single-site study, no evidence of statistically or clinically significant ischemia was seen with the use of ketamine for procedural sedation. Ketamine remains a safe medication option in adults undergoing procedural sedation.
